# Supplementary material for: Antibacterial and Photocatalytic Activity of ZnO/Au and ZnO/Ag Nanocomposites
Source: Int J Mol Sci. 2023 Nov 29;24(23):16939. doi: 10.3390/ijms242316939 (PMC10706707; doi:10.3390/ijms242316939)
Supplement: Supplementary file 1 [file ijms-24-16939-s001.zip › ijms-2710947-supplementary.pdf]

Article

Antibacterial and photocatalytic activity of ZnO/Au and ZnO/Ag nanocomposites

Mariana Busila [1,*], Viorica Musat [1], Petrică Alexandru [1], Cosmin Romanitan [2], Oana Brincoveanu [2], Vasilica Tucureanu [2], Iuliana Mihalache [2], Alina Viorica Iancu [3,4], and Violeta Dediu [2,*]

[1] Centre of Nanostructures and Functional Materials-CNMF, Engineering Faculty, "Dunarea de Jos" University of Galati, Domneasca Street 111, 800201, Galati, Romania
[2] National Research and Development Institute in Microtechnologies – IMT Bucharest, 126A Erou Iancu Nicolae Street, 077190, Bucharest, Romania
[3] Department of Morphological and Functional Sciences, Faculty of Medicine and Pharmacy, "Dunărea de Jos" University, 800008 Galati, Romania
[4] Medical Laboratory Department, Clinical Hospital for Infectious Diseases "Sf. Cuvioasa Parascheva", 800179 Galati, Romania
* Correspondence: mariana.busila@ugal.ro (M. Busila) & violeta.dediu@imt.ro (V. Dediu)

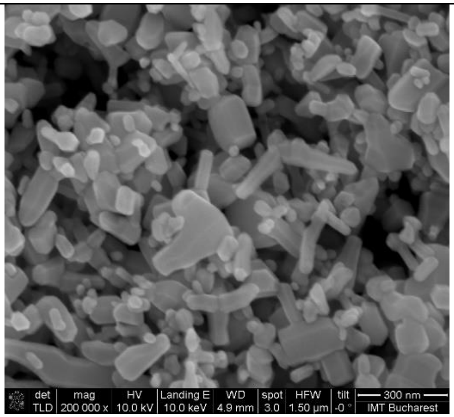	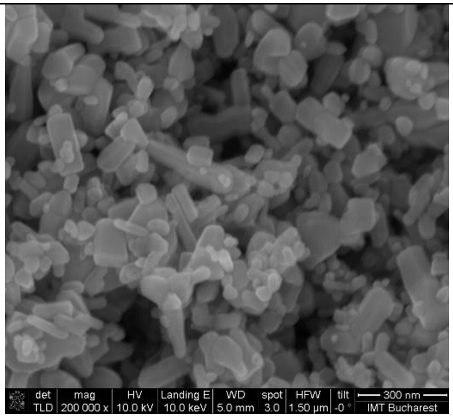	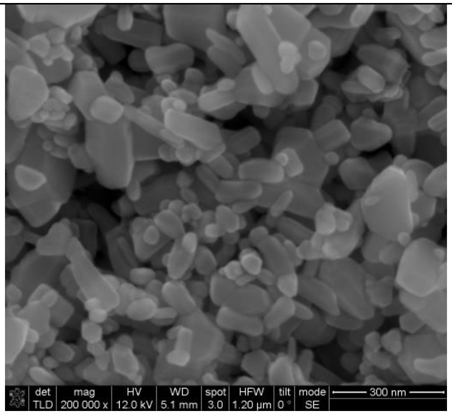	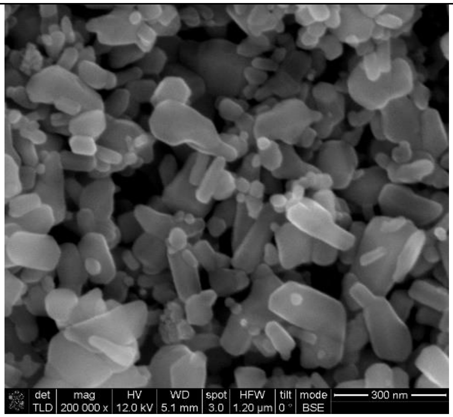
**ZnO comercial(<100nm)**	**ZP1(ZnO-citrate)**	**ZP2(ZnO-citrate+AuNP)**	**ZP3(ZnO-citrate+AgNP)**
**Lenght elongated NPs:**<br>Mean = 131.6 +/42.4 nm, N =150<br>Min – Max = 56 – 286 nm<br>84 – 150 nm<br><br>**Round NPs:**<br>Mean = 43.1+/-15.2 nm, N =250<br>Min – Max = 19 – 118 nm<br>27 – 58 nm	**Lenght elongated NPs:**<br>Mean = 199.5 +/87.2 nm, N =150<br>Min – Max = 88 – 835 nm<br>121 – 231 nm<br><br>**Round NPs s:**<br>Mean = 41.0+/-11.5 nm, N =250<br>Min – Max = 19 – 90 nm<br>28 – 47 nm	**Lenght elongated NPs:**<br>Mean = 190.3 +/64.1 nm, N =150<br>Min – Max = 91 – 599 nm<br>123 – 255 nm<br><br>**Round NPs:**<br>Mean = 48.8+/-17.6 nm, N =250<br>Min – Max = 19 – 127 nm<br>29 – 59 nm	**Lenght elongated NPs:**<br>Mean = 190.3 +/68.1 nm, N =150<br>Min – Max = 102 – 491 nm<br>123 – 226 nm<br><br>**Round NPs:**<br>Mean = 40.4+/-13.7 nm, N =250<br>Min – Max = 15 – 122 nm<br>29 – 59 nm

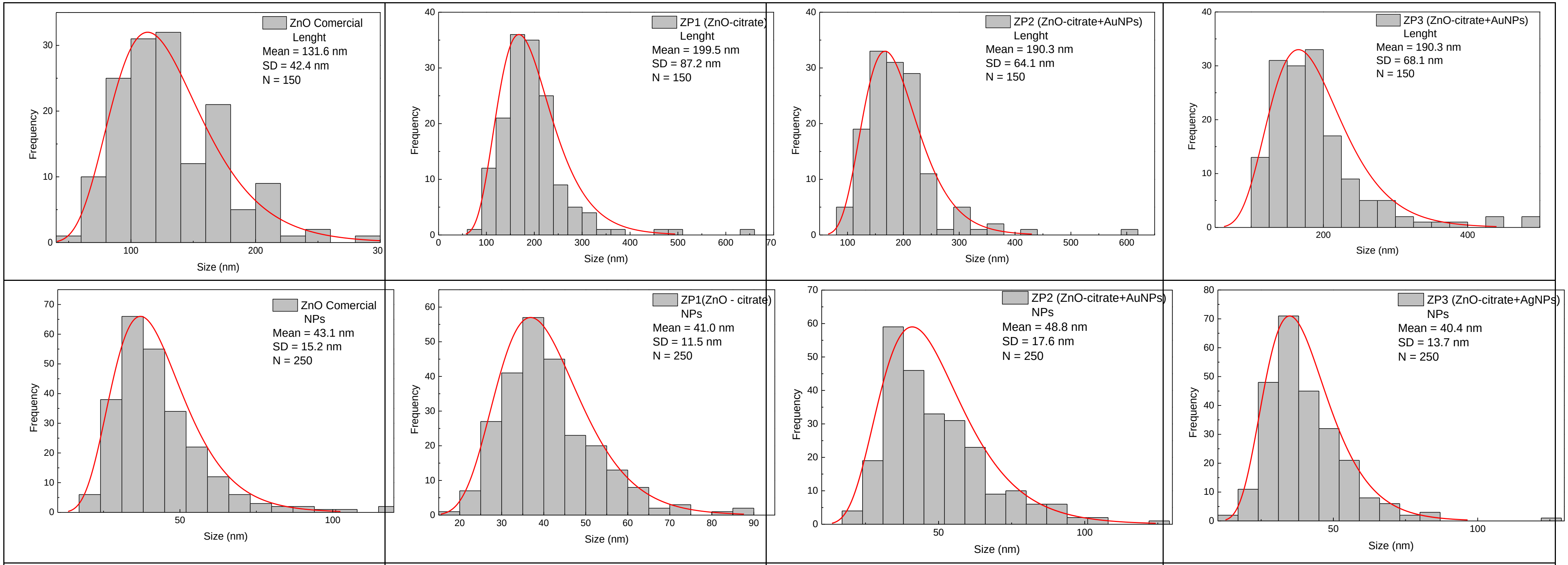

Overall = 76.1 +/51.6 nm, N =400<br>Min – Max = 19 – 286 nm<br>21 – 82 nm	Overall = 101.1 +/94.5 nm, N =400<br>Min – Max = 19 – 835 nm<br>20 – 141 nm	Overall = 99.4 +/79.1 nm, N =400<br>Min – Max = 19 – 599 nm<br>24 – 191 nm	Overall = 97.3 +/84.1 nm, N =400<br>Min – Max = 19 – 599 nm<br>24 – 191 nm

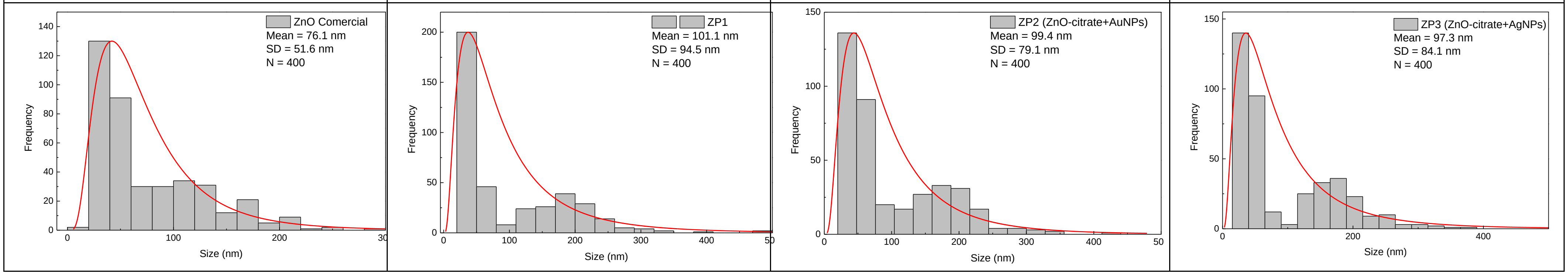

Figure S1 Statistical analysis of nanoparticles dimensions using ImageJ

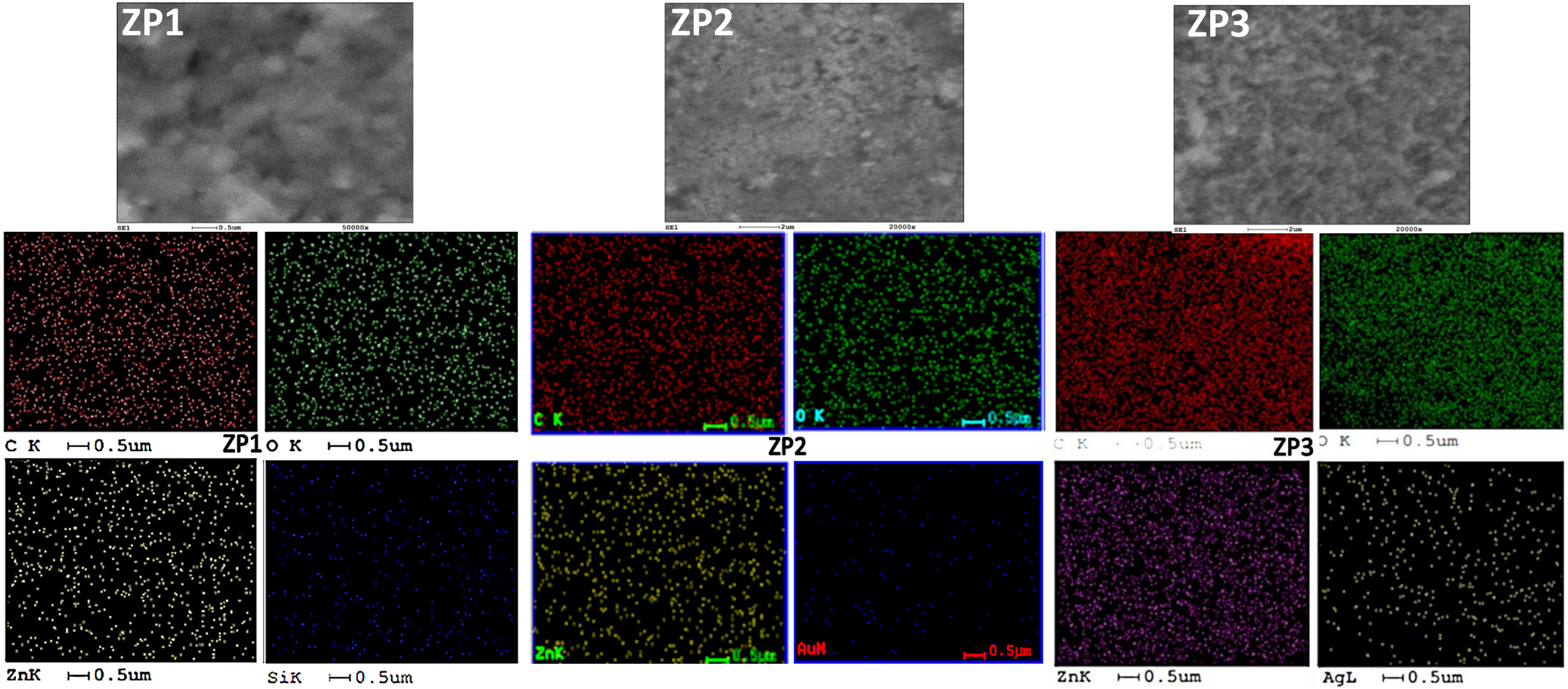

Figure S2. EDS elemental maping of ZP1, ZP2, and ZP3 samples

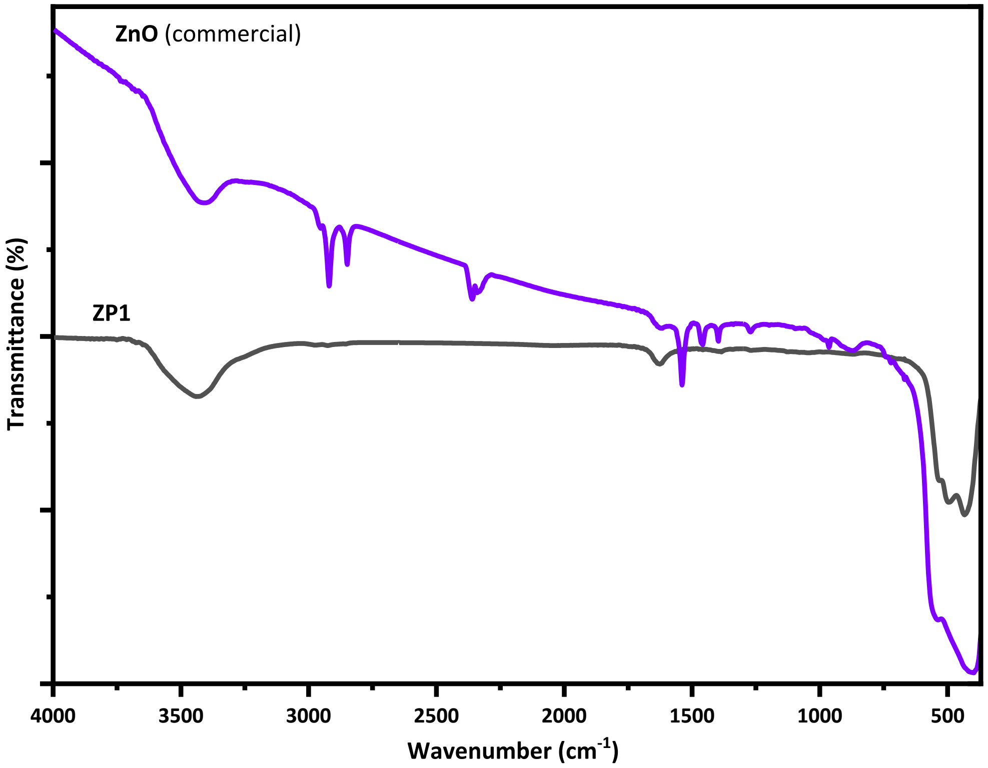

Figure S3. FTIR spectra for commercial ZnO and ZP1 samples

As can be seen from Figure S2 certain bands assigned to organic dispersant disappeared from the ZP1 spectrum.
